# Non-coding RNA and autophagy: Finding novel ways to improve the diagnostic management of bladder cancer

**DOI:** 10.3389/fgene.2022.1051762

**Published:** 2023-01-04

**Authors:** Ishaq Tantray, Rani Ojha, Aditya P. Sharma

**Affiliations:** ^1^ School of Medicine, Department of Pathology, Stanford University, Stanford, CA, United States; ^2^ Department of Urology, Postgraduate Institute of Medical Education and Research (PGIMER), Chandigarh, India

**Keywords:** non-coding RNA, autophagy, bladder cancer, drug resistance, miRNA and lncRNA

## Abstract

Major fraction of the human genome is transcribed in to the RNA but is not translated in to any specific functional protein. These transcribed but not translated RNA molecules are called as non-coding RNA (ncRNA). There are thousands of different non-coding RNAs present inside the cells, each regulating different cellular pathway/pathways. Over the last few decades non-coding RNAs have been found to be involved in various diseases including cancer. Non-coding RNAs are reported to function both as tumor enhancer and/or tumor suppressor in almost each type of cancer. Urothelial carcinoma of the urinary bladder is the second most common urogenital malignancy in the world. Over the last few decades, non-coding RNAs were demonstrated to be linked with bladder cancer progression by modulating different signalling pathways and cellular processes such as autophagy, metastasis, drug resistance and tumor proliferation. Due to the heterogeneity of bladder cancer cells more in-depth molecular characterization is needed to identify new diagnostic and treatment options. This review emphasizes the current findings on non-coding RNAs and their relationship with various oncological processes such as autophagy, and their applicability to the pathophysiology of bladder cancer. This may offer an understanding of evolving non-coding RNA-targeted diagnostic tools and new therapeutic approaches for bladder cancer management in the future.

## Introduction

Urothelial carcinoma of the urinary bladder is the second most commonly diagnosed urogenital cancer (Saginala et al., 2020; Compérat et al., 2022). On initial presentation, 70–75% of patients with bladder tumors are categorized as non-muscle invasive bladder cancer (NMIBC). The initial approach for the management of NMIBC is cystoscopy followed by transurethral resection of the tumor. Approximately 47% of patients have disease recurrence within 5 years of diagnosis of NMIBC and 9% of patients may progress to muscle-invasive disease. At present, bladder cancer management is limited to surgery, chemotherapy, radiotherapy, and immunotherapy. Personalized medicine may offer new windows for the management of bladder cancer (Lemke and Shah, 2018; Pardo et al., 2020). The next-generation sequencing (NGS) technology has discovered extensive tumor heterogeneity in different cancer types. Furthermore, RNA sequencing revealed upregulation of non-coding RNA (ncRNA) specifically microRNA, and long non-coding RNA (lncRNA) in bladder cancer (Cong et al., 2019). The human genome is 98% transcribed, however, most of them do not encode proteins, and are reported as ncRNAs (Pop-Bica et al., 2017; Cong et al., 2019). ncRNAs have been demonstrated to regulate gene expression as well as transcription, splicing, and protein function (Pop-Bica et al., 2017; Liu et al., 2022a). Recently, lncRNAs have been shown to regulate autophagy (Ghafouri-Fard et al., 2022). Autophagy is a conserved catabolic process in which intracellular components are engulfed and degraded, serving various functions depending upon the cellular conditions (Onorati et al., 2018; Ojha et al., 2019; Ishaq et al., 2020). Under normal conditions, it protects the cells from starvation by recycling damaged proteins and organelles. On the other hand, defective autophagy in a cell predisposes it to cancer (Ojha et al., 2015). Autophagy has been shown to help in tumor survival and progression by reducing ROS levels and providing nutrients (Ojha et al., 2014; Ishaq et al., 2016; Ojha et al., 2019). However, the role of autophagy in cancer is very complex and has been reviewed in detail somewhere else (Poillet-Perez et al., 2015; Amaravadi et al., 2019). Role of lnc-RNA in tumor progression *via* autophagy modulation is actively being investigated. In a recent study by Tan et al. showed that a set of seven autophagy related lnc-RNAs may predict the disease prognosis in MIBC patients (Tan et al., 2022). Similarly, Lai et al. set of 15 lnc-RNA related with autophagy were found to predict the prognosis of bladder cancer. Using the bioinformatics, these authors found that the 15-autophagy related lnc-RNA may regulate bladder cancer progression by modulating cell cycle, cell adhesion, DNA replication and WNT signalling pathways (Lai et al., 2020).

In this review, we will discuss the applicability of ncRNA in the progression and development of bladder cancer. We review the regulation of autophagy by ncRNA, especially miRNA and lncRNA, and the contribution of the ncRNA-autophagy axis in inducing tumor growth and/or tumor suppression in bladder cancer. Also, we will focus on the potential of exploring ncRNA expression profiles for the diagnosis and treatment of bladder cancer.

## Bladder cancer and non-coding RNAs (ncRNAs)

Bladder cancer is one of the most commonly diagnosed malignancies of the urinary tract and is characterized by low sensitivity to chemotherapy and a high recurrence rate (Saginala et al., 2020; Compérat et al., 2022). Low-grade bladder cancer has a slow progression rate and hardly presents a threat to patients. On the other hand, high-grade bladder cancer has malignant potential with a greater mortality rate (Berdik, 2017; Daneshmand, 2020). Chemical agents, physical stimuli, and some pathogens are among the common factors responsible for the conversion of normal urothelial cells to the malignant urothelium. 95% of the primary urothelial tumors are confined within the bladder (Siracusano et al., 2020). Patients with this disease need lifelong surveillance because of the high recurrence rate, particularly if the tumor is within the bladder. Therefore, bladder cancer is the most expensive cancer to manage and because of this financial burden, bladder cancer is currently a very important focus of research (Audenet et al., 2018). Almost 60–65% of all bladder cancer show loss of heterozygosity on chromosome 9 and approximately 40% of bladder cancer show loss of heterozygosity on chromosome 17. Loss of heterozygosity on chromosome 9 is believed to be among the initial events in bladder carcinogenesis (Anderson, 2018). In contrast, loss of heterozygosity on chromosome 17 occurs late during cancer development and is related to the aggressiveness of cancer. Gene encoding p53 (TP53) is located on chromosome number 17 and most of the bladder cancer show loss of one allele of 17p resulting in loss of tumor suppressor functions of p53 (Li et al., 2021a). Mutations in the retinoblastoma (tumor suppressor) gene have been also observed in muscle-invasive, high grade and also in superficial bladder cancers (Li et al., 2021a). Bladder cancer cells have a higher expression of anti-apoptotic proteins Bcl-2 and Bcl-xL and the increased expression of these anti-apoptotic proteins correlates with poor prognosis in bladder cancer patients (Real and Malats, 2007). Bcl-2 overexpression in bladder cancer has been also reported to play an important role in cisplatin resistance (Cho et al., 2006). The very first study by Zhong et al. has shown atypical/aberrant expression of ncRNAs in bladder cancer might be exploited as bladder cancer biomarkers (Zhong et al., 2019).

**FIGURE 1:**
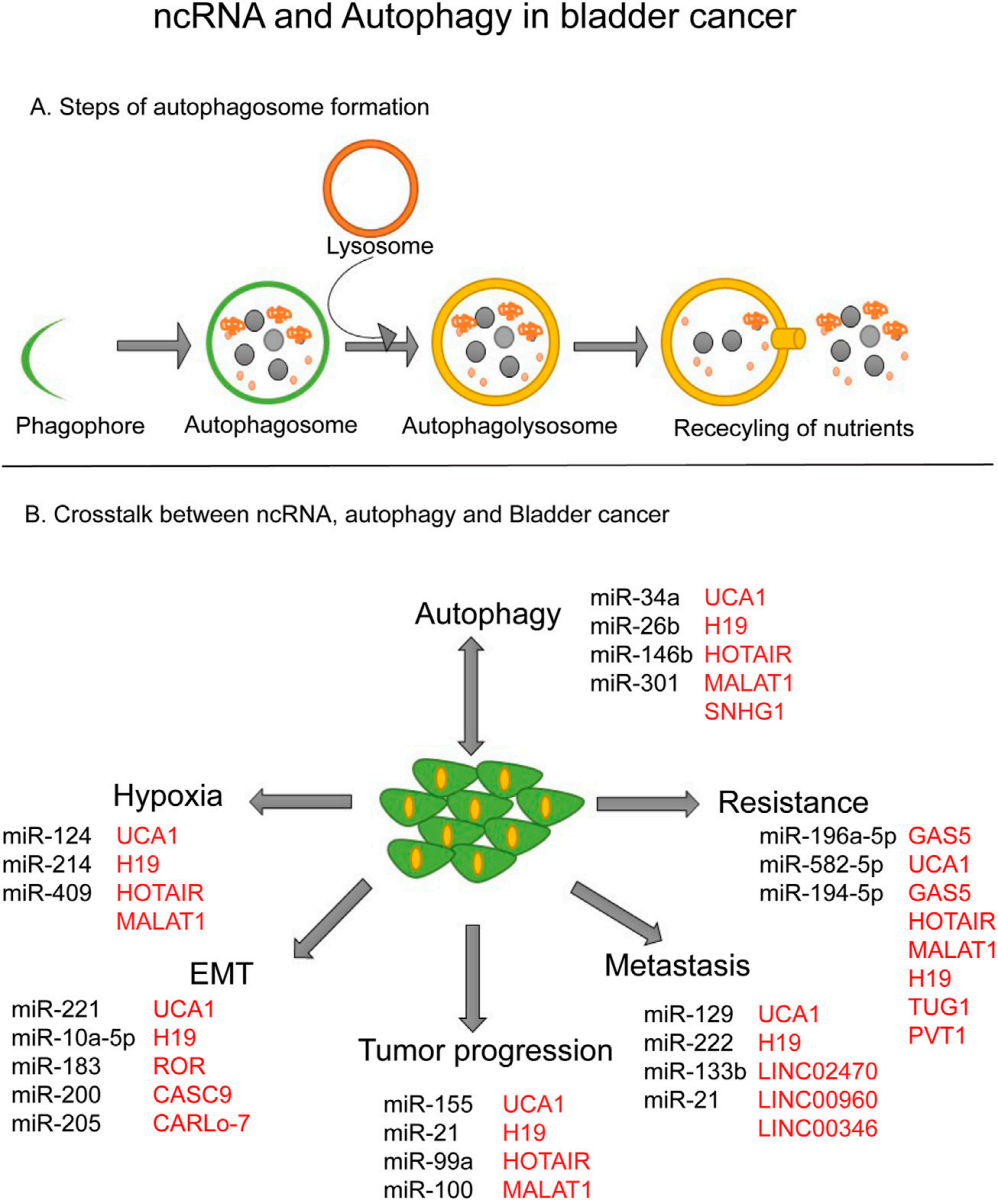
ncRNA and Autophagy in bladder cancer. **(A)** Steps of autophagosome formation. **(B)** Crosstalk between ncRNA, autophagy and bladder cancer.

High-throughput next-generation sequencing has shown that the human genome is approximately 90% transcribed. However, the transcribed genome can encode only 20,000 proteins (Reuter et al., 2015). A non-coding RNA (ncRNA) is a functional RNA molecule that is not translated into a protein. Emerging evidence suggests that ncRNAs are fundamental players in the regulation of gene expression and play a key role in various pathological conditions (Churko et al., 2013). Additionally, ncRNAs have been shown to mediate mRNA degradation, remodeling of chromatin structure, and also in cis and trans gene regulation (Fernandes et al., 2019; Ning et al., 2019). There are mainly two types of ncRNA characterized by their length: small ncRNAs and long ncRNAs (lncRNAs). Small ncRNAs include ribosomal RNA (rRNA), transfer RNA (tRNA), microRNA (miRNA), circular RNA, and piwi-interacting RNA (piRNA) (Zhang et al., 2019a; Papaioannou et al., 2021). lncRNAs include intergenic lncRNAs, intronic ncRNAs, and sense and antisense lncRNAs. Each type of lncRNAs showing different genomic positions in relation to genes and exons. lncRNAs regulate protein translation and post-transcriptional levels (Statello et al., 2021). Circular RNAs are different from linear mRNAs, however exact roles and functions are not fully understood yet (Cech and Steitz, 2014). In the following sections, we will be focusing on the role of miRNA and lncRNAs in different oncological processes in tumor bladder cancer (Figures 1A, B).

## miRNA and bladder cancer

**FIGURE 2:**
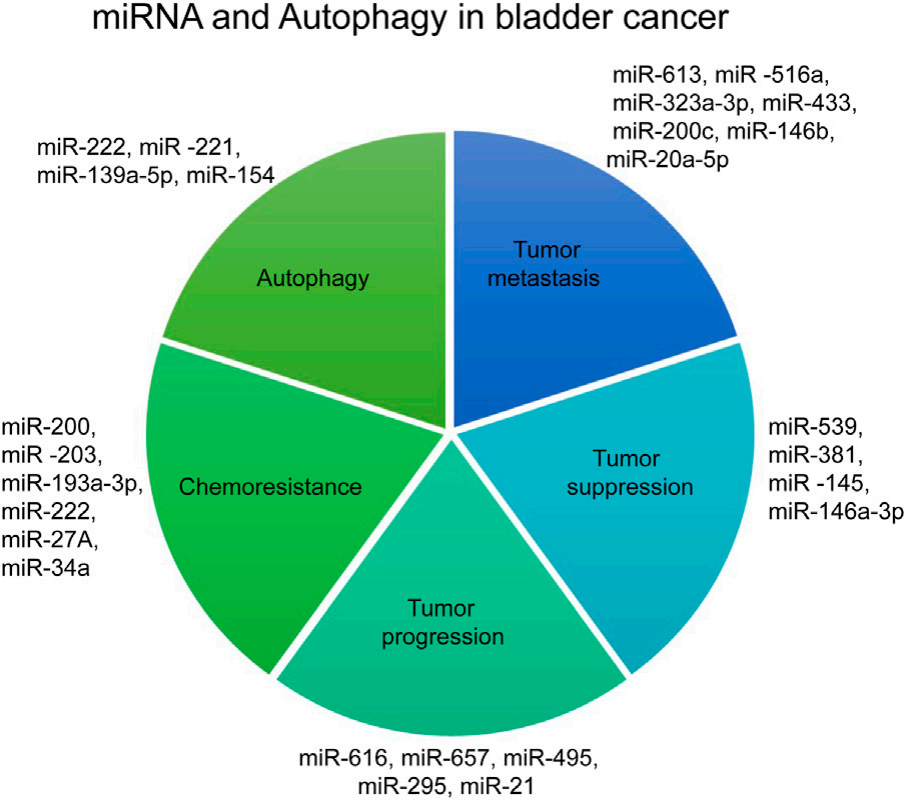
miRNA and Autophagy in bladder cancer.

miRNAs are single-stranded ncRNAs with a length of nearly 22 nucleotides (O’Brien et al., 2018). miRNAs are responsible for regulating gene expression at the post-transcriptional level, thereby modulating cellular processes like growth, proliferation, differentiation, and apoptosis (Towler et al., 2015). The miRNAs are produced from the conversion of pri-miRNA to pre-miRNA by a protein complex consisting of Rosha and the double-stranded RNA binding protein DiGeorge Syndrome Critical Region 8 (DGCR8). Pre-miRNAs are around 65 nucleotide-long RNA intermediates in the nucleus and are transported to the cytoplasm by Exportin-5. After the transportation, the pre-miRNA is cleaved by DICER and integrated into an RNA-induced silencing complex (RISC). When miRNA is complementary to the 3′UTR region of target mRNA, RISC will inhibit the protein translation and mRNA will be degraded by endonucleases (Michlewski and Cáceres, 2019; Saliminejad et al., 2019; Rani and Sengar, 2022). The biology of miRNA processing and mechanism of action have been explored well and is reviewed by Statello et al. (Statello et al., 2021). Here we will be mainly focusing on the role of miRNAs in various hallmarks of bladder cancer (Figure 2).

### Tumor progression

MiR-616 is located on chromosome 12q13.3 and has been shown to be upregulated in many cancers (Zhao et al., 2022). Upregulated expression of miR-616 was shown to increase bladder cancer cell progression by targeting SOX7 (SRY-related HMG-box). SOX7 is a transcription factor belonging to the SRY-related high mobility group (HMG) box family and is known to function as a tumor suppressor. miR-616 downregulates the SOX7 expression by targeting its mRNA for degradation. Inhibition of miR-616 decreased bladder cancer cell proliferation abilities, induced cell cycle arrest in the G2 phase, and enhanced apoptosis (Zhao et al., 2022). MiR-495 is encoded by a gene located on chromosome 14 (q32.31). miR-495 has been linked to various tumors, however, its tumorigenic role is controversial. In bladder cancer, overexpression of miR-495 increased bladder cancer proliferation (Tan et al., 2017). MiR-657 expression was found higher in bladder cancer tissue samples compared to the normal bladder. Overexpression of miR-675 has been reported to promote bladder cancer progression by promoting cell cycle progression. Inhibition of miR-675 decreased p53 activation and increased Bax/Bcl2 ratio and thereby inducing apoptosis in bladder cancer cells (Liu et al., 2016). A high expression of miR-294 was demonstrated in bladder cancer cell lines. Mechanistically, authors have shown that miR-294 upregulates NRAS expression to promote bladder cancer progression. Additionally, overexpression of miR-294 increased the phosphorylation of PI3K, AKT, JNK, and STAT1 (Li et al., 2017a). An upregulated expression of MiR-21 was demonstrated in bladder cancer tissue. MiR-21 overexpression has been shown to increase Bcl2 expression and enhance Akt phosphorylation to promote cell proliferation in bladder cancer cells (Lin et al., 2020).

### Tumor metastasis

miR-516a is located on chromosome 19q13 and has been shown to be involved in different oncogenic processes. miR-516 expression directly correlated with the invasive properties of bladder cancer cells. miR-516a promotes metastasis by regulating the expression of Matrix metallopeptidase 9 (MMP9). miR-516a decreases Surfeit locus protein 1 (SUMRF1) expression thereby stabilizing and inhibiting the degradation of MMP9 by the proteasomal pathway (Chang et al., 2020). Also, another study by Zhu et al. has shown that miR-146b is upregulated in bladder cancer tissues. Silencing of miR-146b inhibited bladder cancer cell invasion by reducing MMP2 level expression by inhibiting its transcription factor ETS2. Mechanistically, authors have demonstrated that miR-146b inhibition stabilizes ARE/poly(U)-binding/degradation factor 1 mRNA expression by directly binding to its mRNA at 3′ UTR (Zhu et al., 2019). Overexpression of miR-200c in bladder cancer cell line UMUC3 decreases their invasive properties. The high expression of miR-200c resulted in increased expression of E-cadherin and decreased expression of Zinc finger E-box-binding homeobox 1 and 2 (ZEB1) and ZEB2 at both mRNA and protein levels. These authors showed that ZEB1 and ZEB2 are the direct targets of miR-200 (Liu et al., 2022b). Recently Yang et al., 2021, showed that miR-20a-5p expression is highly upregulated in bladder cancer tissues. Overexpression of miR-20a-5p not only increased bladder cancer cell proliferation but also promoted epithelial-to-mesenchymal transition (EMT). The overexpression of miR-20a-5p inhibited the expression of epithelial markers (E-cadherin) and increased the Vimentin expression (mesenchymal marker) (Yang et al., 2021). This study showed that miR-20a-5p selectively targets Nuclear Receptor Subfamily four Group A Member 3 (NR4A3) and decreases its expression. So, by targeting this nucleus receptor protein NR4A3-miR-20a-5p regulates the migration, invasion, and metastasis of bladder cancer cells (Yang et al., 2021). On the contrary, there are some miRNAs such as miR-433, miR-323a-3p, and miR-613 that have inhibitory effects on EMT. miR-433 expression was shown to be downregulated in bladder cancer tissue samples. Overexpression of miR-433 decreased migratory and EMT properties of bladder cancer cells by modulating the c-MET-Akt pathway. MiR-433 directly binds to c-MET and CAMP responsive element binding protein 1 (CREB1) at 3′-UTR and inhibits its mRNA as well as protein expression (Xu et al., 2016). The miR-323a-3p level was found to be downregulated in bladder cancer tissue samples as well as in cell lines. These authors demonstrated that the two key oncogenes Suppressor of Mothers against Decapentaplegic 3 (SMAD3) and MET are the direct targets of miR-323a-3p. Also, miR-323a-3p upregulation was shown to inhibit EMT progression in bladder cancer cells by targeting the AKT- Glycogen synthase kinase-3β (GSKB) axis (Li et al., 2017b). Just like miR-323a-3p, the expression of miR-613 is downregulated in bladder cancer cell lines and tumor tissues. miR-613 overexpression abrogated EMT *via* increasing the expression of E-cadherin and decreasing that of Vimentin and Snail. miR-613 was found to exert its inhibitory effects on EMT *via* blocking the expression of Sphingosine kinase 1 (Sphk1) (Yu et al., 2017a).

### Chemoresistance

The recurrence rate in bladder cancer is of common occurrence particularly due to the cancer cells developing resistance towards standard drugs like gemcitabine, cisplatin, *etc.* (Lemke and Shah, 2018). There are various mechanisms by which cancer cells develop resistance towards any anti-cancer drug, but recently microRNAs have been also added to that list (Taheri et al., 2020). The role of miRNAs in drug resistance was first studied by Fojo et al., who showed that miRNA profile can play a key role in chemosensitivity or chemoresistance (Fojo, 2007). In bladder cancer, the first identified drug-resistance-related miRNAs belonged to the miR-200 family. MiR-200 overexpression in bladder cancer cells increases their sensitivity towards the epidermal growth factor receptor (EGFR) inhibitors *via* regulating the expression of ERBB receptor feedback inhibitor 1 (ERRF1) (Adam et al., 2009). MiR-21 has been shown to be highly upregulated in bladder cancer cells and contributes to doxorubicin resistance. MiR-21 enhanced resistance towards doxorubicin was mediated by increased expression of anti-apoptotic protein Bcl2 (Tao et al., 2011). miR-203 was associated with cancer progression and poor prognosis of bladder cancer patients who received cisplatin-based adjuvant chemotherapy (Zhang et al., 2015). In contrast to miR-21, miR-203 expression is highly reduced in bladder cancer patients. Overexpression of miR-203 in bladder cancer cells increased their sensitivity to cisplatin by inducing apoptosis. In their study, Zhang et al. showed that two pro-apoptotic proteins, Bcl-w and Survivin are the direct targets of miR-203 (Zhang et al., 2015). MiR-193a-3p has been found to be more highly expressed in resistant cell lines than in sensitivity cell lines. Interestingly, miR-193a-3p was shown to promote multi-drug resistance in bladder cancer cells. The mechanism by which miR-193a-3p enhanced drug resistance was shown to be the inhibition of Serine/arginine-rich splicing factor 2 (SRSF2) and lysyl oxidase-like 4 (LOXL4) expression in bladder cancer cells (Deng et al., 2014). In a very interesting study, miR-218 was reported to enhance the cisplatin sensitivity of bladder cancer cells by targeting Glut1 (glucose transporter isoform 1). Glut1 expression was shown to be significantly decreased in miR-218 overexpressed cells. This is very important; because restricting the availability of glucose during chemotherapy has been shown to enhance drug sensitivity in different cancer models (Li et al., 2017c). MiR-222 increases resistance towards cisplatin by regulating Protein Phosphatase 2 Regulatory Subunit Balpha (PPP2R2A)/Akt/mTOR pathway. PPP2R2A is a regulatory subunit of phosphatase 2A and is known to play a role in cancer progression. One of the direct targets of PPP2R2A is Akt. In fact, the overexpression of miR-222 was shown to increase the Akt phosphorylation and activates its downstream target mTOR (Zeng et al., 2016). miR-27A is downregulated in bladder cancer tissue samples and is involved in enhancing cisplatin resistance by regulating the expression of Solute Carrier Family 7 Member 11 (SLC7A11). SLC7A11 is highly overexpressed in cells that are resistant to cisplatin. SLC7A11 is a component of cysteine/glutamate exchanger and is an important factor regulating glutathione (GSH) production. Overexpression of miR-27A significantly decreases SLC7A11 expression and enhances sensitivity to bladder cancer cells toward cisplatin (Drayton et al., 2014). In a recent study, CD44 has been shown to be targeted by miR-34a in muscle-invasive bladder cancer during cisplatin treatment. An increased expression of CD44 has been shown to effectively reverse the effects of miR-34a on bladder cancer cell proliferation and chemosensitivity of muscle invasive-bladder cancer cells (Li et al., 2022a).

### Autophagy

Autophagy is an umbrella process for the degradation of aggregated proteins and organelles including mitochondria, endoplasmic reticulum, ribosomes, and nucleus (Dikic and Elazar, 2018). The contribution of autophagy in cancer development is well established although still controversial (Ojha et al., 2015; Ishaq et al., 2020). The role of autophagy is highly dependent on the cancer type, mutational status, and developmental stage of cancer (Onorati et al., 2018; Amaravadi et al., 2019). The regulatory networks involved in autophagy induction and/or inhibition have been well characterized. MiRNAs are the latest addition to the long list of autophagy regulators (Xu et al., 2012; Shan et al., 2021). Inhibition of autophagy *via* selective targeting of Beclin1 by miR30a was the first report, highlighting the role of miRNAs in autophagy and cancer (Zhu et al., 2009). The role of miRNAs in autophagy regulation was first discovered in 2009 when Beclin1 (BECN1), an autophagy gene, was shown to be regulated by miR30A (Cai et al., 2021). miR-221 induces cell proliferation of colorectal cancer (CRC) *via* the downregulated of autophagy by targeted Tumor protein 53-induced nuclear protein 1 (TP53INP1) (Liao et al., 2018a).

Autophagy has been associated with both chemoresistance and chemosensitivity (Li et al., 2019a). MiR-222 has been shown to inhibit cisplatin-induced cell death by preventing autophagy induction. MiR-222 inhibits autophagy induction *via* the activation of the Akt/mTOR signaling axis. mTOR is one of the key negative regulators of autophagy involved in cancer progression (Tsikrika et al., 2018). Tsikrika et al. have reported that bladder cancer patients with high expression of miR-221 have a higher short-term recurrence rate. Moreover, miR-221 overexpression has also been reported to be an independent prognostic value for bladder cancer patients (Tsikrika et al., 2018). Liu et al. have shown that the downregulation of miR-221 enhances autophagy activation *via* increasing TP53INP1. In addition, the miR-221/TP53INP1/p-ERK axis has been shown to regulate autophagy in bladder cancer (Liu et al., 2020a). miR-24-3p is also overexpressed in bladder cancer cells and has been shown to promote autophagy by inhibiting domain-containing protein (DEDD). Autophagy induction *via* miR-24-3p helped bladder cancer cell proliferation by apoptosis inhibition (Yu et al., 2017b). Luo et al. have shown that miR-139-5p inhibited bladder cancer cell proliferation by direct binding Bmi-1. Inhibition of Bmi-1 has been demonstrated to increase ATP reduction and AMPK-activated autophagy (Luo et al., 2017). Wang et al. has reported that sodium butyrate inhibited bladder cancer cell migration and induced AMP-activated protein kinase (AMPK)-mTOR axis-dependent autophagy and ROS-mediated apoptosis *via* the miR-139-5p/Bmi-1 pathway (Wang et al., 2020). Interestingly, Zhang et al. have shown that miRNA-mediated autophagy downregulation has been shown to inhibit bladder cancer cell progression. miR-154 has been shown to downregulate bladder cancer tissue samples. Overexpression of miR-145 inhibits bladder cancer cell proliferation, invasion, and migration by preventing ATG7 expression (Zhang et al., 2019b).

In, multiple myeloma (MM), miR-126 mediates the induction of autophagic flux and HIF1α stabilization (Tomasetti et al., 2016). In bladder cancer, miR-221 expression was shown to be positively regulated by TGFβ1. Inhibition of miR-221 rescued TGFβ1-induced EMT by increasing E-cadherin and decreasing vimentin, Fibronectin, and N-cadherin. Moreover, miR-221/TP53INP1/p-ERK axis expression was shown to induce autophagy and reported to be positively correlated with the malignant property of bladder cancer cells (Shen et al., 2021). The level of miR‐133b was found to be reduced in bladder cancer patient tissues and in exosomes from the serum of bladder cancer patients. Exosomal miR‐133b leads to the inhibition of cancer cell viability by upregulating dual‐specificity protein phosphatase 1 (DUSP1) and an escalation of apoptotic cell death in bladder cancer cells (Cai et al., 2020). Small nucleolar RNA host gene 1 (SNHG1) is another lncRNA (lncRNA), which negatively regulates tumor suppressor genes. Increased expression of SNHG1 has been shown to enhance bladder cancer progression and autophagy *via* miR-493-5p/ATG14/autophagy pathway (Guo et al., 2021). This finding highlights the potential role of SNHG1 as a target for the management of bladder cancer. miR-21 shows a grade-dependent increase in bladder cancer cells. This study has demonstrated that, in 31 patients, miR-21 was significantly up-regulated, and PTEN level was significantly inhibited in bladder tumor tissue compared to the normal bladder mucosa (Yin et al., 2019). Furthermore, patients with recurrence had a significantly higher miR-21 expression as compared to non-recurrent patients. Also, miR-21 overexpression has been reported to decrease autophagy and promote the metastatic properties of bladder cancer cells (Zhang et al., 2020). This study is interesting because we have also shown that autophagy induction is grade dependent in bladder cancer (Ojha et al., 2014). The grade dependency of miR-21 and autophagy may be unrelated but needs to be experimentally verified.

### Tumor suppression

miR-381 was shown to be downregulated in bladder cancer. Increased expression of miR-381 was demonstrated to inhibit the proliferation and tumor formation capacity of bladder cancer cell lines T24 and RT4 cells. In addition, miR-381 was found to bind B cell-specific Moloney murine leukemia virus integration site 1 (BMI1). The tumor-suppressing properties of miR-381was shown to be blocked by overexpression of BMI. Overexpression of miR-381 decreased RhoA phosphorylation and Rho-associated protein kinase (ROCK2) activation (Chen et al., 2021a). miRNA-145 was significantly downregulated in patients with bladder cancer. miRNA-145 markedly inhibited the ability of bladder cancer cells to migrate and invade. Furthermore, N-cadherin was identified as a target of miRNA-145 in bladder cancer cells. MMP9, acting downstream of N-cadherin, was downregulated in bladder cancer cells by miRNA-145 (Zhang et al., 2018). The expression of miR-146a-3p was found to be downregulated in bladder cancer tissue samples. MiR-146a-3p overexpression has been shown to downregulate the metastatic potential of bladder cancer cells. MiR-146a-3p directly binds to 3′UTR of an oncogene Pituitary tumor-transforming gene 1 (PTTG1) (Xiang et al., 2017). A decreased expression of miR-539 was reported in bladder cancer. Overexpression of miR-539 inhibits bladder cancer cell proliferation. This study showed that IGF1R is a direct target of miR-539. The mimic of miR-539 decreased IGF1R expression *via* binding to its 3′UTR region. In addition, authors have shown that silencing of miR-539 attenuated the phosphorylation of AKT and ERK. This leads to the inhibition of bladder cancer growth and invasion by the AKT-ERK-IGF1R axis (Liao et al., 2018b).

## Long non-coding RNA and bladder cancer

**FIGURE 3:**
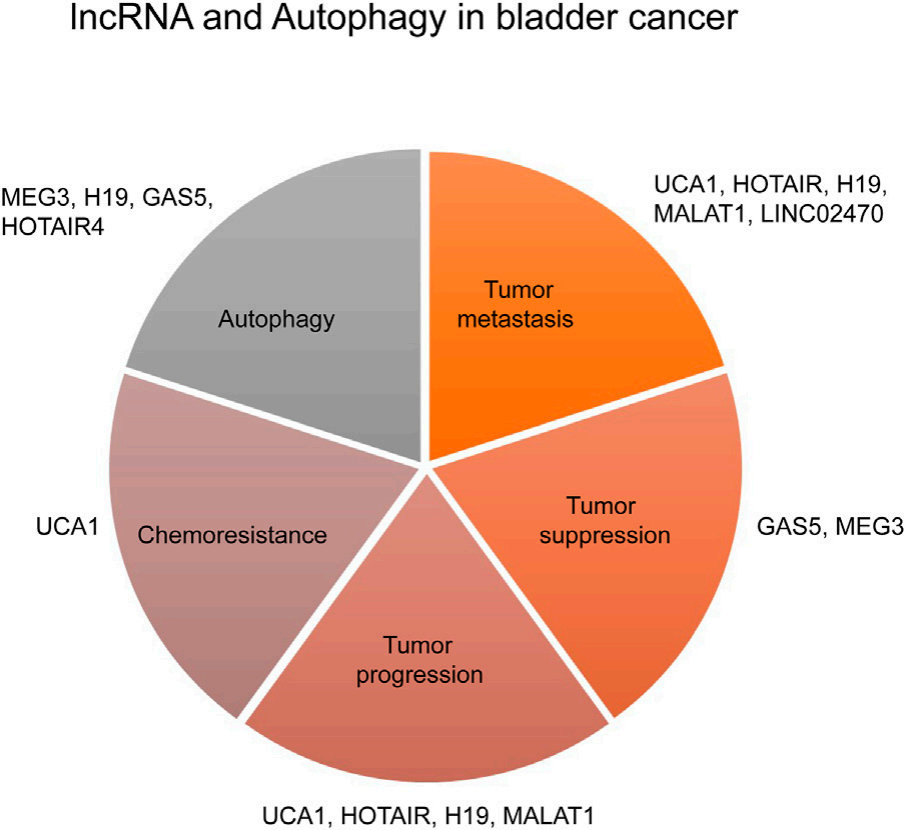
IncRNA and Autophagy in bladder cancer.

Long non-coding RNAs (lncRNAs) are RNA molecules of more than 200 nucleotides that are not translated. LncRNAs are transcribed by RNA Polymerase-II and are mainly localized to the nucleus. Although lncRNAs are abundant in the nucleus, they are less stable than their cytoplasm-localized counterparts. LncRNAs are stabilized either by polyadenylation or by the formation of secondary structure triple helices at their 3′ end. These 3′end sequences help in the nuclear export of lncRNAs (Nojima and Proudfoot, 2022). LncRNA expression is usually low but shows stronger tissue-specific expression, indicating lncRNAs play an integral role in tissue-specific processes (Aznaourova et al., 2020). Various studies have reported the role of lncRNAs in carcinogenesis as well as other pathologies (Yao et al., 2019a). The role of lncRNA in bladder cancer progression, proliferation, and metastasis has been shown in several studies (Zhang et al., 2021a; Chen et al., 2021b; Li et al., 2021b). In the following section, we will discuss the role of lncRNAs in different oncogenic processes in bladder cancer (Figure 3).

### Tumor progression

Urothelial cancer associated 1 (UCA1) is one of the lncRNA associated with bladder cancer progression. UCA1 is located on chromosome 19p13.12 (Mirzaei et al., 2022). The ectopic expression of UCA1 influences bladder tumor progression, revealing UCA1 as an oncogenic player in bladder carcinogenesis (Ding et al., 2021). The role of UCA1 in bladder cancer tumorigenesis has been reported in various studies (Ghafouri-Fard et al., 2022). UCA1 has been demonstrated to increase EMT by zinc finger E‐box binding homeobox 1 and 2 (ZEB1 and ZEB2) upregulation in bladder cancer cells (Xue et al., 2016). UCA1 is known to be transported *via* exosomes and leads to bladder cancer progression under hypoxic conditions (Xue et al., 2017). In a contrast study, Lebrun et al. showed that in a particular type of bladder cancer UCA1 expression is decreased (Lebrun et al., 2018).

Homeobox transcript antisense RNA (HOTAIR) genes reside in the HOXC cluster, located on the human chromosome 12q13.1 (Tang and Hann, 2018). HOX cluster is an important key factor in embryonic development. Suppression of the HOX genes has been demonstrated in tumor progression (Gupta et al., 2010). HOTAIR inhibited p53 expression and the phosphatase and tensin homolog (PTEN) expression, thereby aggravating cancer progression (Zhang et al., 2019c). HOTAIR expression also significantly correlates with the activation of the Wnt/β-catenin pathway (Zhang et al., 2021b). H19 is one of the first discovered ncRNAs, which is located at chromosome 11p15.5. H19 is highly expressed during human embryonic development but is suppressed in adults (Wu et al., 2021). However, H19 has been shown to reactivate during tumorigenesis and play a crucial role in various cancer including bladder cancer (Raveh et al., 2015; Ghafouri-Fard et al., 2020). H19 has been shown to enhance bladder cancer cell proliferation *via* the upregulation of an inhibitor of DNA binding 2 (ID2) (Luo et al., 2013a). The ID2 upregulation attenuates retinoblastoma protein (Rb) effects on E2F1 expression, thereby promoting bladder cancer progression (Mao et al., 2021). Similarly, H19 has been shown to induce upregulation of p53 protein thereby leading to bladder cancer progression (Atala, 2013). Metastasis-associated lung adenocarcinoma transcript 1 (MALAT1) has been first identified as the prognostic marker in lung cancer and has been reported to have a positive association with many other cancers including prostate cancer and hepatic cancer (Amodio et al., 2018; Sun and Ma, 2019). The overexpression of MALAT1 enhances bladder cancer progression and migration (Xie et al., 2017a; Li et al., 2017d; Liang et al., 2021). Additionally, MALAT1 has been shown to inhibit apoptotic cell death and thereby promote bladder cancer progression.

### Tumor Metastasis

UCA1 by regulating the high mobility group box 1 (HMGB1) pathway enhances the properties of bladder cancer invasion and metastasis. HMGB1, a member of the high mobility group box subfamily, has been demonstrated to be associated with various cancer (Wu and Zhou, 2018). HMGB1 acts as an EMT inducer in many human cancer cells (Dong et al., 2022) and is significantly upregulated higher in bladder cancer cells than in normal urothelial cells (Hajjari and Salavaty, 2015). Several studies have reported that HOTAIR is the main player in various malignancies including bladder cancer, colon cancer, lung cancer, and breast cancer (Chi et al., 2019). Liu et al. showed that silencing of HOTAIR inhibits the invasive properties of bladder cancer by downregulating epithelial-to-mesenchymal (EMT), suggesting HOTAIR’s role in regulating metastasis (Liu et al., 2015). In addition, the inhibition of HOTAIR has been demonstrated to regulate Notch1-mediated EMT pathways in bladder cancer and thereby promoting metastatic properties of bladder cancer (Berrondo et al., 2016).

In addition, H19 upregulated expression has been demonstrated to play a vital role in bladder cancer metastasis. Authors have shown that H19 overexpression increased the migratory properties of bladder cancer cells by interaction with EZH2 which leads to activation of Wnt/β-catenin signaling and therefore led to EMT induction (Atala, 2013; Luo et al., 2013b). Zhu et al. has also shown that H19 overexpression leads to enhancing the metastatic properties *via* EMT pathways (Zhu et al., 2018). The expression of MALAT1 was shown to be higher in invasive and metastatic bladder cancer compared to normal tissue. Additionally, MALAT1 expression was higher in high-grade patients compared to low-grade bladder cancer patients (Li et al., 2017d). Silencing of MALAT1 has an inhibitory effect on the metastatic properties of bladder cancer cells. MALAT1 downregulates ZEB1, ZEB2, and Wnt signaling proteins, while it upregulates E-cadherin (Ying et al., 2012; Fan et al., 2014a). These results indicate that MALAT1 has a key role in initiating metastatic properties in bladder cancer. Recently, LINC02470 was reported to enhance bladder cancer cell viability, migration, and invasion. LIN02470 was demonstrated to activate the SAMD3-TGF β-mediated EMT process in bladder cancer (Huang et al., 2022). Similarly, Haung et al. has shown that two exosome-derived lncRNAs; LINC00960 and LINC02470 increased the malignant properties of bladder cancer cells by upregulating EMT, β-catenin, NOTCH, and SMAD signaling in high-grade bladder cancer (Huang et al., 2020).

### Chemoresistance

There are only a few reports where lncRNAs have been shown to facilitate drug resistance in bladder cancer (Liu et al., 2020b). The overexpression of UCA1 was shown to abrogate apoptotic cell death and facilitate acquired resistance during anti-cancer therapy in bladder cancer cells (Wang et al., 2017). This study has demonstrated that increased overexpression of UCA1 decreased the sensitivity of tamoxifen and silencing of UCA1 mediates/enhances drug sensitivity by induction of apoptosis and cell cycle arrest during tamoxifen treatment (Liu et al., 2019). Mechanistically, UCA1 was shown to physically interact with EZH2, which blocked the p21 expression through histone methylation, and parallelly UCA1 expression mediates phosphorylation of CAMP responsive element binding protein (CREB) and PI3K/AKT and thereby facilitates tamoxifen-mediated resistance (Fan et al., 2014b). In addition, UCA1 has also been shown to mediate resistance during cisplatin treatment by Cytochrome P450 Family 1 Subfamily B Member 1 (CYP1B1)-mediated apoptosis *via* miR-513a-3 upregulation (Li et al., 2022b). UCA1 expression was shown to be positively related to CYP1B1 expression. UCA1 binds with miR-513a-3p to induce CYP1B1 expression. UCA1 silencing promotes chemosensitivity and enhances apoptosis during cisplatin treatment, suggesting the UCA1/miR-513a-3p/CYP1B1 axis plays a key role in mediating chemoresistance (Cheng et al., 2021). The role of UCA1 in colorectal cancer cells (CRC) has also been studied. A study by Liu et al. demonstrated that the UCA1-Wnt/β-catenin axis in CRC plays an imperative role in regulating metastasis and autophagy *in vivo* (Liu et al., 2019).

### Autophagy

Stress is a common feature of tumors, which include hypoxia, and deficiency of nutrient growth factors due to insufficient vasculature (Hanahan, 2022; Ojha et al., 2022). Autophagy is one of the main cellular processes which is induced in many tumors under various stress conditions like hypoxia and starvation (Mulcahy Levy and Thorburn, 2020). For the first time, our group (Ojha et al.) showed that autophagic flux increases in a grade-dependent manner in bladder cancer. AMPK is the key factor for regulating autophagy in bladder cancer cells and functionally autophagy plays a cytoprotective role in these cells (Ojha et al., 2014). Moreover, inhibition of autophagy by both pharmacological and siRNA enhanced the chemotherapeutic effects in bladder cancer cells (Ojha et al., 2016). Gambogic acid, a potent anticancer agent, also has been shown to induce autophagic flux by reactive oxygen species (ROS) mediated JNK activation in the bladder cancer cells (Ishaq et al., 2014).

Autophagy has been demonstrated to be regulated by several mechanisms in cancer cells. Among the known mechanisms, the recently validated mechanism is ncRNAs (Yang et al., 2017). Several miRNAs and lncRNAs have been described to regulate autophagy *via* distinct mechanisms (Yao et al., 2019b). Various genetic and biochemical studies have illustrated that the inhibition of MEG3 expression enhances autophagy and blocks apoptosis (Xiu et al., 2017). A study by Ying et al. has shown that MEG3 expression was markedly reduced in bladder cancer compared with normal bladder tissues, however, an increased autophagy level was significantly found in tumor tissue compared to normal bladder tissue. Inhibition of MEG3 expression blocked apoptosis and increased cell proliferation while autophagy inhibition augmented MEG3-silencing-mediated apoptosis (Ying et al., 2013). These findings accentuate the significance of MEG3 in tumor suppression and the potential use of targeting MEG3 in the management of bladder cancer. A study by Xiu et al., 2017 has demonstrated that upregulated expression of MEG3 inhibited tumorigenesis *in vivo* by upregulating ATG3 expression (Xiu et al., 2017). In addition, MEG3 has been shown to protect ATG3 mRNA from degradation during actinomycin D treatment. P53 activation has been demonstrated to regulate MEG3 expression to protect against tumor proliferation (Xiu et al., 2017). MEG3 overexpression has been shown to downregulate miR-96 while upregulating α-tropomyosin 1 (TPM1), which reduced bladder cancer cell viability by increasing apoptotic cell death (Liu et al., 2018).

The overexpression of H19 was found to inhibit GTP-binding protein Di-Ras3 (DIRAS3) expression and induce phosphorylation of mTOR, which leads to the inhibition of autophagy in cardiomyocytes (Zhuo et al., 2017). Treatment of bladder cancer cells with exosomes isolated from tumor-associated macrophages enhances H19 expression and autophagic response. Inhibition of H19 in TAMs-exosomes blocked autophagy flux (Guo et al., 2022). This study suggests that H19 plays a role in autophagy induction in bladder cancer cells and targeting TAMs-Exosomes-H19 is an encouraging therapeutic approach for the management of bladder cancer. Overexpression of GAS5 induced chemoresistance to cisplatin, which was not rescued by 3-MA-mediated inhibition of autophagy, signifying that GAS5 promotes chemosensitivity in an autophagy-independent manner (Xu et al., 2020). Silencing of GAS5 decreased cancer cell viability and reduced autophagy *via* regulating miR-23a expression (Li et al., 2018). The results suggested that the GAS5-miR-23a complex might be involved in the regulation of autophagy. Collectively, these results indicated that GAS5 participates in carcinogenesis by stimulating the autophagy response. To the best of our knowledge of GAS5 and autophagy in bladder cancer is not studied yet. HOTAIR was shown to increase autophagy and promote imatinib sensitivity of gastrointestinal stromal tumors (GIST). This study has shown that miR-130a and HOTAIR have downstream target autophagy-related protein 2 homolog B (ATG2B). Downregulation of ATG2B blocks the effect of HOTAIR on imatinib sensitivity in GIST (Zhang et al., 2021b). In addition, inhibition of HOTAIR has been demonstrated to enhance the sensitivity to radiotherapy by inhibiting autophagy through the downregulation of the Wnt signaling pathway in cervical cancer (Trujano-Camacho et al., 2021). To the best of our knowledge, there are no reports about the role of HOTAIR in regulating autophagy in Bladder cancer.

### Tumor suppressor

Growth arrest-specific 5 (GAS5) was initially shown to regulate cell proliferation and cell cycle during embryogenesis and tumor progression (Yu and Hann, 2019; Xu et al., 2020). However, several studies have demonstrated that the downregulation of GAS5 is potentiating tumor progression in various cancers including bladder cancer (Cao et al., 2016). Similarly, GAS5 has been demonstrated as a tumor suppressor role in bladder cancer *via* inhibiting EZH2 expression and augmenting apoptosis (Kaur et al., 2022). GAS5 has been also shown to inhibit Calcium-Activated Chloride Channel 1 (CCLA1) expression and thus contributes to the suppression of cancer growth (Jia et al., 2015). Additionally, genetic studies have demonstrated that GAS5 suppresses cell division protein kinase 6 (CDK6), thereby inhibiting bladder cancer progression (Wang et al., 2012). Furthermore, GAS5 has been reported to promote apoptosis by suppressing EZH2 transcription *via* the recruitment of transcription factor E2F4 to EZH2 promoter in bladder cancer cells (Cao et al., 2016). Altogether, these studies suggest that GAS5 could use for bladder cancer patient’s treatment. However, GAS5 silencing was reported to reduce apoptosis *via* glucocorticoid receptors during nutrient deprivation (Kino et al., 2010).

Maternally expressed gene 3 (MEG3) is highly expressed in human tissue and has been demonstrated to play as a tumor suppressor (He et al., 2017). MEG3 expression has been shown to inhibit bladder cancer progression (He et al., 2017). Ying et al. have reported that MEG3 expression was downregulated in bladder cancer compared with normal tissue (Liu et al., 2018). Further genetic studies demonstrated the silencing of MEG3 increases autophagy and abrogated apoptosis *in vitro* (Ying et al., 2013). These studies underscore the significance of MEG3 in tumor suppression in bladder cancer treatment.

## Clinical relevance of non-coding RNA

The clinical use of ncRNAs has recently gained momentum because of some advantages over the currently used drugs. First, ncRNAs are synthesized by the cells themselves, so will not be treated as alien molecules. Second, ncRNAs usually target a set of different mRNAs, which encode proteins regulating a specific process. However, so far only one ncRNA, Prostate Cancer Associated 3 (PCA3) is used in clinical settings as a biomarker for prostate cancer. Various ncRNAs are under clinical trials for different pathologies including cancer. However, the effective use of ncRNAs in therapeutics is limited because of issues like; delivery to target regions, bioavailability, and specificity (Wang et al., 2019; Winkle et al., 2021). In the following section we will be highlighting the clinical relevance of ncRNAs in bladder cancer.

### Clinical utility of miRNA: Diagnosis and prognosis

Various biomarker studies have demonstrated the potential of miRNAs in bladder cancer diagnosis, for example high expression of the miR-200 family correlated with better overall and recurrence-free survival (Table 1). Mei et al. concluded that high expression of the miR-200 family is strongly associated with better prognosis in bladder cancer patients and may significantly improve bladder cancer management (Mei et al., 2020). Similarly, higher expressions of hsa-miR-663a and hsa-miR-3648, and lower expression of hsa-miR-185-5p, hsa-miR-30c-5p, hsa-miR-1270, hsa-miR-200c-3p, and hsa-miR-29c-5p, significantly correlated with shorter overall survival of bladder cancer patients (Homami and Ghazi, 2016; Lin et al., 2019). A combination of 7 miRNAs (7-miRNA panel: miR-6087, miR-6724-5p, miR-3960, miR-1343-5p, miR-1185-1-3p, miR-6831-5p and miR-4695-5p) was shown to accurately discriminate bladder cancer from non-cancer and other types of tumors with the high specificity (Usuba et al., 2019). Another study showed that the combination of four miRNAs; miR-181b-5p, miR-183-5p, miR-199-5p and miR-221-3p can be used as a stable biomarker for bladder cancer diagnosis (Li et al., 2022c).

**TABLE 1:** miRNAs in bladder cancer.

Name of miRNA	Target	Signaling pathways	Function	Level	Clinical significance
miR-10b	-----	------	Tumor progression	Increased	Diagnostic, prognostic
miR21	P53, AKT, PTEN, Maspin, VEGF-C	PI3K/AKT, and miR-21/maspin/VEGFC pathway	Apoptosis, and mesenchymal transition, promote tumor progression	Increased	Diagnostic, prognostic
miR-23a	FOXO3, IL6R	PI3K-AKT signaling pathway	Cancer Progression	Decreased	Therapeutic target
miR-124	ROCK1	-------	Migration and invasion	Decreased	Diagnostic, prognostic
miR-126	ADAM9	------	Tumor suppressor	Increased	Therapeutic target
miR129	GALNT1 and SOX4	------	Provide drug resistance	Increased	Diagnostic, prognostic
miR133b	BCl-w	Akt	Tumor suppressor	Increased	Diagnostic, prognostic
miR-141-3p	PTEN	Cell cycle	Metastasis	Increased	Diagnostic, prognostic
miR-145	FSCN1	EMT inhibition	Tumor suppressor	Decreased	Therapeutic target
miR-155	DMTF1	DMTF1-Arfp53	Tumor progression		
miR-200	-----	ZEB1 and ZEB2	EMT and metastasis	Decreased	Diagnostic, prognostic
miR-205-5p	ZEB1, ZEB2, PTEN, AKT, VEGF	Adherent junctions, focal adhesion	tumorigenesis, invasion and metastasis	Increased	Prognostic, therapeutic target
miR210	E2F3, FGFRL1, HOXA1	------	Cell growth, migration, apoptosis	Increased	Diagnostic, prognostic
miR221	-----	JAK-STAT	Bladder tumor progression	Increased	Diagnostic, prognostic
miR-222	-----	------	Associated with unfavorable clinical features and poor survival	Increased	Diagnostic, prognostic
miR409	c-Met and c-Fos	------	Migration and invasion	Decreased	Prognostic
miR-590-3p	TFAM, (PI3K), AKT, MMP2 and MMP9	-------	Tumorigenesis	Decreased	Therapeutic target

Recent studies showed circulating miRNA by liquid biopsy could be the potential biomarker for bladder cancer. Mitesh et al. found miR-7-5p, miR-22-3p, miR-29a-3p, miR-126-5p, miR-200a-3p, miR-375, and miR-423-5p in urine could serve as non-invasive biomarkers for bladder cancer (Mitash et al., 2017). Lian et al. found miR-148b-3p, miR-3187-3p, miR-15b-5p, miR-27a-3p, and miR-30a-5p in serum samples could be the potential biomarkers for the prognosis of bladder cancer (Lian et al., 2018). A study by Xie et al. identified seven miRNAs were up-regulated in bladder serum cancer samples compared to control samples, including hsa-miR-185-5p, hsa-miR-663a, hsa-miR-30c-5p, hsa-miR-3648, hsa-miR-1270, hsa-miR-200c-3p, and hsa-miR-29c-5p (Xie et al., 2017b; Li et al., 2022c). The dysregulation of these miRNAs correlated with the advanced stage and overall survival in bladder cancer patients.

### Clinical utility of long non-coding RNA: Diagnosis and prognosis

Just like miRNAs, lncRNAs have been explored as possible diagnostic biomarkers for tumor bladder cancer (Table 2). UCA1 is among the well-studied and good candidates for bladder cancer biomarkers. UCA1 mRNA expression shows a significant association with the stage and grade of bladder cancer (Lebrun et al., 2018). Yu et al. have shown increased expression of UCA1 in urine samples of high-grade bladder tumor patients (Yu et al., 2020). UCA1 has been shown to have high sensitivity for the T2-T4 stage of bladder cancer, suggesting that UCA1 may be considered a marker for the better prognosis of bladder cancer (Avgeris et al., 2019). Expression of lncRNAs H19 and HOTAIR are significantly increased in bladder cancer tissues compared to normal bladder tissues (Wang and Yin, 2017). High expression of HOTAIR and GAS5 was shown to be associated with poor survival in bladder cancer (Li et al., 2019b). A study by Lodewijk et al. has demonstrated that UCA1, HOTAIR, and MALAT1, can be used as urinary biomarkers for bladder cancer patients (Lodewijk et al., 2018). Another study showed that overexpression of HOTAIR, MALAT1 and LINC00477 was found in urine exosomes of high-grade muscle-invasive bladder cancer patients. Irregular expression of HOTAIR, UBC1, H19, and GAS5 has been reported to be related to overall survival in bladder cancer (Berrondo et al., 2016) however, more confirmative studies are needed to make a definite conclusion. HOTAIR expression was shown to be associated with prognosis, and poor disease-free survival in bladder cancer (Heubach et al., 2015). Another study by Yan et al. demonstrated a correlation between HOTAIR high expression with histological grade and recurrence rate (Zhang et al., 2022). Additionally, HOTAIR was also shown to be enriched in urinary exosomes of high-grade muscle-invasive bladder cancer (Berrondo et al., 2016). Another lncRNA GAS5 was found to be decreased in bladder cancer. Downregulated expression of GAS5 was shown to be related to poor disease-free survival (Cao et al., 2016). H19 expression was also found to correlate with overall survival (Luo et al., 2013b). MALAT1 overexpression was reported to have a significant association with the grade and metastasis in bladder cancer (Amodio et al., 2018), indicating that MALAT1 may be used as a prognostic biomarker in bladder cancer.

**TABLE 2:** Summary of lncRNAs used as diagnostic and prognostic biomarkers of bladder cancer.

LncRNA	Expression level	Target	Function	Clinical parameters	Clinical utility
UCA1	Upregulated	BMP9/pAKT/UCA1, miR-1/UCA1	Tumor progression and Metastasis	Tumor metastasis	TNM staging
		UCA1/mTOR-STAT3/HK2			
		UCA1/CREB/miR-196a-5p			
		UCA1/BRG1/p21			
		C/EBPα/UCA1, UCA1/(CDKN2B)			
H19	Up-regulation	H19/miR-29b-3p/DNMT3B	Tumor progression	Tumor size	Diagnosis, Prognosis
		H19/miR-675/p53			
HOTAIR	Upregulation	HuR/HOTAIR/miR-1	Resistance	Tumor recurrence	Diagnosis, prognosis
		HOTAIR/miR-205/Cyclin J			Therapeutic target
MALAT1	Upregulation	MALAT1/miR-124/foxq1	Tumor progression	Tumor size	Diagnosis, Prognosis
DUXAP8	Up-regulation	DUXAP8/PTEN	Tumor progression	Tumor size	Diagnosis, Prognosis
TUG1	Up-regulation	TUG1/HMGB1	Tumor progression	Tumor size	Diagnosis, Prognosis
		TUG1/miR-29c, miR-142/ZEB2			
DUXAP8	Upregulation	DUXAP8/PTEN	Metastasis	Tumor metastasis	TNM staging
ZEB1-AS1	Upregulation	ZEB1-AS1/miR-200b/FSCN1	Metastasis	Tumor metastasis	TNM staging
GAPLINC ZFAS1 NORAD SNHG5 SNHG16 PCAT-1 MALAT1 CAT266 CAT1297 CAT1647 UBC1 LSINCT5	Upregulation	Not known	Tumor progression	Tumor size	Diagnosis, Prognosis
CDKN2B-AS PVT1 SNHG16 Linc00857 FOXD2-AS1	Upregulation	Not known	Tumor metastasis	Tumor size	Diagnosis, Prognosis
MEG3	Downregulation	MEG3/miR-96/TPM1	Tumor suppression	Tumor size	Diagnosis and prognosis
		MEG3/miR-494/PTEN			
		MEG3/LC3-I/II			
		MEG3/miR-27a/PHLPP2			
		TP73-AS1/(vimentin			
		MMP-2/9, snail			
		E-cadherin)			
CASC2	Downregulation	CASC2/Wnt-catanin	Tumor suppression	Stage and grade of tumor	TNM staging
GAS5	Downregulation	GAS5/miR21/PTEN	Tumor suppression	Drug resistance	Therapeutic target
		GAS5/(CCL1, Bcl2, CDK6)			
TP73-AS1	Downregulation	Not known	Tumor suppression	Drug resistance	Therapeutic target
PTENP1					

## Future perspectives

With advancing research, it has been well established that ncRNAs are linked to tumor progression, metastasis, and chemoresistance (Cho et al., 2006; Real and Malats, 2007). Several clinical studies have indicated that upregulated or downregulated expression of ncRNAs is associated with the grade, stage, and metastatic potential of cancer cells. Therefore, ncRNAs can be utilized for the early detection and better management of bladder cancer (Real and Malats, 2007). However, the functional role of ncRNAs is still perplexing due to the intricacy and multiplicity of their expression profiles and targeting proteins. Both miRNA and lncRNAs interact with mRNAs which affect different cellular processes like immune response, proliferation, and autophagy (Ning et al., 2019). The effects of ncRNAs as autophagy inducers or inhibitors have been studied; however, there is a prerequisite for more experimental studies in preclinical and clinical settings. It is worth noting that autophagy plays a dual role in cancer, acting not only as a tumor suppressor but also tumor enhancer process. Therefore, the relationship between autophagy and ncRNAs may not be simple. The effect of autophagy inhibitors in combination with targeted knockdown or overexpression of specific ncRNAs on bladder cancer progression needs to be explored. Blocking autophagy has been shown to sensitize the bladder cancer cells to gemcitabine and cisplatin. In the future, ncRNAs which are strong inhibitors of autophagy can be used in combination with these standard drugs to increase their efficacy in bladder cancer patients. Since autophagy is a key process in developing chemoresistance, targeted inhibition of autophagy by ncRNAs may sensitize these cells toward the standard chemotherapeutic drugs. The diagnostic, therapeutic, and prognostic value of the ncRNA and autophagy in urologic cancers needs to be further investigated for the better treatment and management of bladder cancer. In addition to specific proteins and the corresponding signaling pathways, some other factors, such as tumor microenvironment and tumor immunity also play roles in the development of urologic cancers. The role of ncRNAs in these processes will be really interesting to study. The molecular mechanism of the ncRNA-autophagy-immunity axis and its role in urologic tumorigenesis must be further clarified and explored. The answer to these questions may be helpful for finding new diagnostic biomarkers and innovative therapeutic alternatives in urologic malignancies.

## Conclusion

Various studies have clearly established the role of ncRNAs in tumor progression, mediating metastatic potential and drug resistance in bladder cancer. Also, the role of ncRNAs as diagnostic and prognostic markers for bladder cancer has been demonstrated by several studies. Once the role of ncRNAs is well explored, the therapeutic strategies can be precisely tailored for the effective treatment of bladder cancer. For example, by targeting a specific ncRNA the sensitivity towards routine drugs can be significantly increased. In addition, the combinatorial intervention of targeting both ncRNAs and autophagy might be a favorable therapeutic strategy for bladder cancer. With the help of new cutting-edge technologies, the role of ncRNA will be crucial for the development of precision medicine.
